# Author Correction: Ultra-high-resolution observations of persistent null-point reconnection in the solar corona

**DOI:** 10.1038/s41467-023-38149-6

**Published:** 2023-04-25

**Authors:** X. Cheng, E. R. Priest, H. T. Li, J. Chen, G. Aulanier, L. P. Chitta, Y. L. Wang, H. Peter, X. S. Zhu, C. Xing, M. D. Ding, S. K. Solanki, D. Berghmans, L. Teriaca, R. Aznar Cuadrado, A. N. Zhukov, Y. Guo, D. Long, L. Harra, P. J. Smith, L. Rodriguez, C. Verbeeck, K. Barczynski, S. Parenti

**Affiliations:** 1 School of Astronomy and Space Science, Nanjing University, 210093 Nanjing, China; 2 Max Planck Institute for Solar System Research, 37077 Göttingen, Germany; 3 Key Laboratory of Modern Astronomy and Astrophysics (Nanjing University), Ministry of Education, 210093 Nanjing, China; 4 School of Mathematics and Statistics, University of St. Andrews, Fife, KY16 9SS Scotland UK; 5 Sorbonne Université, Observatoire de Paris - PSL, École Polytechnique, IP Paris, CNRS, Laboratory for Plasma Physics (LPP), 4 place Jussieu, 75005 Paris, France; 6 Rosseland Centre for Solar Physics, Institute for Theoretical Astrophysics, Universitetet i Oslo, P.O. Box 1029, Blindern, 0315 Oslo, Norway; 7 State Key Laboratory of Space Weather, National Space Science Center, Chinese Academy of Sciences, Beijing, China; 8 Solar-Terrestrial Centre of Excellence - SIDC, Royal Observatory of Belgium, Ringlaan -3- Av. Circulaire, 1180 Brussels, Belgium; 9 Skobeltsyn Institute of Nuclear Physics, Moscow State University, 119992 Moscow, Russia; 10 Mullard Space Science Laboratory, University College London, Holmbury St. Mary, Dorking, Surrey RH5 6NT UK; 11 PMOD/WRC, Dorfstrasse 33, CH-7260 Davos Dorf, Switzerland; 12 ETH-Zürich, Wolfang-Pauli-Strasse 27, HIT J 22.4, 8093 Zürich, Switzerland; 13 Institut d’Astrophysique Spatiale, Université Paris-Saclay, 91405 Orsay Cedex, France

**Keywords:** Solar physics, Astrophysical plasmas

Correction to: *Nature Communications* 10.1038/s41467-023-37888-w, published online 13 April 2023

In this article the grant number ERC, ORIGIN, 101039844, relating to funding by the European Union for L. P. Chitta was omitted.

The original article has been corrected.

