# Supplementary material for: Ultra-high-resolution observations of persistent null-point reconnection in the solar corona
Source: Nat Commun. 2023 Apr 13;14:2107. doi: 10.1038/s41467-023-37888-w (PMC10102217; doi:10.1038/s41467-023-37888-w)
Supplement: Supplementary file 1 — Supplementary Information [file 41467_2023_37888_MOESM1_ESM.pdf]

Ultra-high-resolution Observations of Persistent Null-point Reconnection in the Solar Corona

Supplementary Information

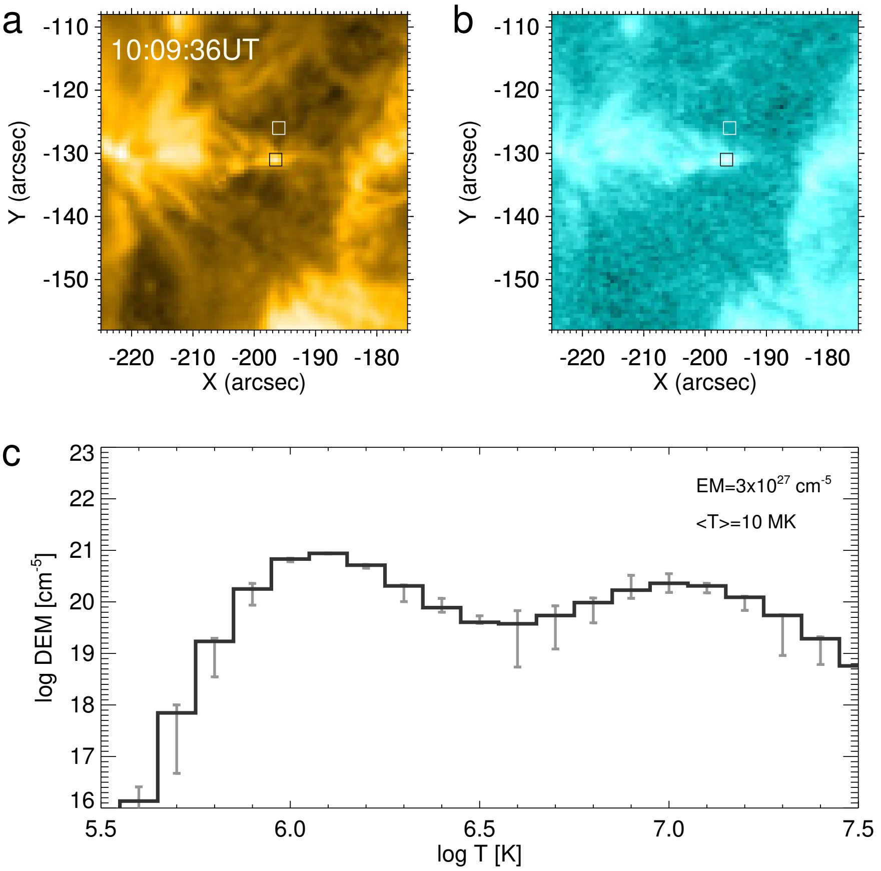

Supplementary Fig. 1: a and b. AIA 171 Å and 131 Å images indicating the null-point configuration at 10:09:36 UT. The point-like brightening and its background (approximated by a nearby quiescent region) are shown by the back and white boxes, respectively. c. DEM curve of the point-like brightening as the function of temperature. The solid curve is the best fitting result directly from observational data. The uncertainties are represented by the error bars, within whose upper and lower limits 90% of MC solutions larger and smaller than the best fitting values are contained, respectively. The total EM and average temperature are integrated in the range of $5.5 \leq \log T \leq 7.4$.

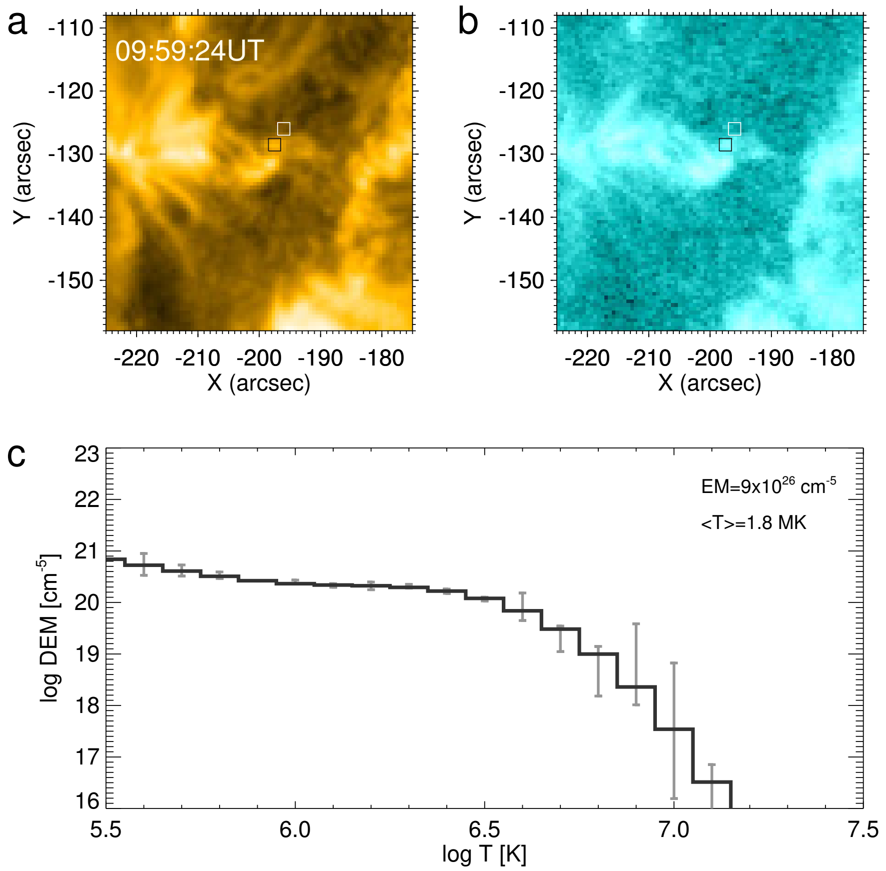

Supplementary Fig. 2: Same as Supplementary Fig. 1 but for an erupting blob from the null-point.
