# Supplementary material for: Ultra-high-resolution observations of persistent null-point reconnection in the solar corona
Source: Nat Commun. 2023 Apr 13;14:2107. doi: 10.1038/s41467-023-37888-w (PMC10102217; doi:10.1038/s41467-023-37888-w)
Supplement: Supplementary file 3 — Description of Additional Supplementary Files [file 41467_2023_37888_MOESM3_ESM.docx]

**Description of Additional Supplementary Files**

**File Name: Supplementary Movie 1
Description:** 174 Å movie of the persistent reconnection at the nullpoint with the duration of about 1 hour observed by SolO EUI/HRIEUV on 2022 March 3.

**File Name: Supplementary Movie 2
Description:** 131 Å, 94 Å, 335 Å, 171 Å, 193 Å, 211 Å movie of the persistent reconnection at the null-point observed by SDO/AIA.

**File Name: Supplementary Movie 3**

**Description:** Movie for temporal evolution of 3D null-point and fan-spine configuration with the duration of 6 hours.

**File Name: Supplementary Movie 4**

**Description:** 171 Å movie of the persistent reconnection with the duration of 5 hours.
